# PERSPECTIVES FROM RETIRED AND EMERITI FACULTY ON STAYING ENGAGED IN HIGHER EDUCATION

**DOI:** 10.1093/geroni/igae098.0987

**Published:** 2024-12-31

**Authors:** Andrea June, Carrie Andreoletti, Helena Swanson

**Affiliations:** Central Connecticut State University, New Britain, Connecticut, United States; Central Connecticut State University, New Britain, Connecticut, United States; Central Connecticut State University, New Britain, Connecticut, United States

## Abstract

With the aim of continuing to strengthen age-inclusive initiatives across Central Connecticut State University, the present study used a survey methodology to explore the interest and ability of retired and emeritus faculty from CCSU to remain engaged with the university. In a preliminary analysis of results, respondents (N=38, M age = 73, 49% female identifying, 67% non-Hispanic White) were mostly full professors (69%) at the time of their retirement. Retirement dates ranged from 1999 to 2024, with an average of 26 years of service. The most common reason for retirement was feeling emotionally ready (31%) followed by feeling burnt out/campus climate issues (26%). Twenty eight percent reported that their retirement was accelerated by the COVID 19 pandemic. Not surprisingly, those whose retirement dates are more recent have remained more connected through teaching and mentorship. At least 50% of respondents felt at least slightly connected to CCSU, their prior department, and current faculty. However, there was greater variation in level of satisfaction with that level of connection. More than half of the respondents expressed support for and willingness to participate in a retired/emeriti professors association at CCSU. Overall, respondents expressed more interest in short, time limited opportunities to remain engaged with students compared to semester-long activities. Qualitative data analysis revealed additional context to the challenges of interacting with the university as a retired or emeriti faculty member as well as to the goals of any continued engagement. Plans to incorporate this data into future age-inclusive initiatives will be discussed.

